# Should Heavy Metals Be Monitored in Foods Derived From Soils Fertilized With Animal Waste?

**DOI:** 10.3389/fpls.2018.00732

**Published:** 2018-06-05

**Authors:** Rafael da Rosa Couto, Jucinei J. Comin, Monique Souza, Felipe K. Ricachenevsky, Marcos A. Lana, Luciano C. Gatiboni, Carlos A. Ceretta, Gustavo Brunetto

**Affiliations:** ^1^Técnico em Agroecologia, Instituto Federal Catarinense, Rio do Sul, Brazil; ^2^Agroecossistemas, Centro de Ciências Agrárias, Universidade Federal de Santa Catarina, Florianópolis, Brazil; ^3^Departamento de Biologia, Agrobiologia, Universidade Federal de Santa Maria, Santa Maria, Brazil; ^4^Biologia Celular e Molecular, Universidade Federal do Rio Grande do Sul, Porto Alegre, Brazil; ^5^Department of Crop Production Ecology, Swedish University of Agricultural Sciences, Uppsala, Sweden; ^6^SUSLAND, Leibniz Centre for Agricultural Landscape Research, Müncheberg, Germany; ^7^Ciência do Solo, Centro de Ciências Agroveterinárias, Universidade do Estado de Santa Catarina, Lages, Brazil; ^8^Ciência do Solo, Departamento de Solos, Universidade Federal de Santa Maria, Santa Maria, Brazil

**Keywords:** heavy metals, animal waste, soil fertilization, residue management, seed contamination

Heavy metals (HM) represent a large group of elements with atomic density >5 g cm^−3^ or atomic number >20 (Saidur et al., 2017), among which some are essential to plants, such as iron (Fe), zinc (Zn), copper (Cu), nickel (Ni), and manganese (Mn). However, HMs may be contaminants and/or pollutants, depending on the concentration in soils.

HMs such as Cu, Zn, Ni, and chromium (Cr) are essential to human beings, and biofortification approaches to improve levels of some elements in plant edible parts are underway (Bouis et al., 2012; Ricachenevsky et al., 2015). However, these HMs may be toxic if accumulated, and may only be ingested in very small quantities (EPA-U.S. Environmental Protection Agency, 1995; FAO-Food Agriculture Organization of the United Nations, 1995; Tchounwou et al., 2012). On the other hand, Pb, Cd, As, and Br are not essential and can be toxic even at low concentrations (Tchounwou et al., 2012). The safe daily intake level for As, Cd, Cr, Cu Ni, Pb, and Zn is 20, 300, 1500, 4, 20, 40, 300 μg kg^−1^ of body weight per day, respectively (EPA-U.S. Environmental Protection Agency, 1993). These levels are based on the degree to which the element may cause disturbance, the capacity of the body to accumulate the element and the weight of the individual who is ingesting it (Abbasi et al., 2013). However, when HMs are ingested for long periods, even at doses considered safe, they can cause harmful effects, known as chronic intoxication (Jorge Mendoza et al., 2017; Li et al., 2017).

The increase in total HM concentration and their chemical forms in soils can occur naturally due to atmospheric deposition, weathering of rocks, and anthropic activities such as mining, deposition of ash from coal burning, application of pesticides in plants, addition of mineral and organic fertilizers, among others (Guilherme et al., 2005). HM accumulation in the soil is typically assessed by indicators such as Geo-accumulation index (Igeo) (Equation 1) (Müller, 1979) and Enrichment Factor (EF) (Equation 2) (Abbasi et al., 2013) that allow the identification of the presence and the intensity of deposition of anthropogenic contaminants in topsoil.

(1) $$\begin{array}{l} Igeo=\log_{2\left(\frac{\left[C_{n}\right]}{1.5}*\left[B_{n}\right]\right)} \end{array}$$

where: *Cn* is the measured concentration in the soil for the metal *n, Bn* is the background value for the metal *n*, and the factor 1.5 is used because of possible variations of the background data due to lithological variations.

(2) $$\begin{array}{l} EF=\frac{\left[\frac{metal}{RE}\right]sample}{\left[\frac{metal}{RE}\right]control} \end{array}$$

where: *RE* is the value of metal, adopted as Reference Element.

High HM concentrations in soils may cause intoxication upon inhalation, contact with the skin, indirect ingestion of soil and intake of fruits, vegetables, grains, and their byproducts (Zheng et al., 2010; Chabukdhara and Nema, 2013; Chen et al., 2016; Jiang et al., 2017). Plants grown in soils contaminated/polluted with HM tend to absorb, accumulate, transport, and redistribute larger amounts of HM. This is likely due to the presence of non-selective essential element transporters. For instance, iron high affinity transporter IRT1 of the model plant *Arabidopsis thaliana*, which is necessary for Fe acquisition under iron deficiency, is known to also transport Zn, Mn, Ni, Co, and Cd, possibly leading to metal toxicity under Fe deficiency (Korshunova et al., 1999; Barberon et al., 2014; Ricachenevsky et al., 2018). In rice, IRT1 might also transport Zn and Cd (Lee and An, 2009). Arsenic uptake is also performed by phosphate transporters (as arsenate) or by silicon transporters (as arsenite), which are not able to distinguish between these elements (Kochian et al., 2015). Thus, non-selective transport leads to accumulation of toxic elements, which might end up accumulating in grains or other harvested parts, and may change nutrient abundance and distribution (Punshon et al., 2018). These agricultural products containing high HM concentration might then be used for human consumption directly or indirectly through the intake of processed foods (Hariri et al., 2015; Avkopashvili et al., 2017).

To assess the risk of ingestion of a particular HM over the life of an individual, it is necessary to consider the period of ingestion. Therefore, indexes have been established to verify the risk that certain elements, such as HMs, could cause to human beings (Abbasi et al., 2013). A few examples of these indexes are the Health Risk Index (HRI), Target Hazard Quotient (THQ) and Target Cancer Risk (TCR) (Equation 5) (EPA-U.S. Environmental Protection Agency, 2010).

(3) $$\begin{array}{l} HRI=\frac{\left(C_{n}xD_{n}\right)}{\left(RfDxBW\right)} \end{array}$$

where: *C*_*n*_, total concentration of the metal in edible plant organ (mg kg^−1^); *D*_*n*_, daily intake (g day^−1^); *BW*, average body weight (kg); *RfD*, reference dose (EPA-U.S. Environmental Protection Agency, 2010).

(4) $$\begin{array}{l} HQ=\frac{\left(C_{n}xD_{n}x10^{-3}xEF_{r}xED_{tot}\right)}{RfDxBW_{a}xAT_{n}} \end{array}$$

where: *EF*_*r*_, exposure frequency (days); *ED*_*tot*_, exposure duration (years); *AT*_*n*_, average exposure time to non-carcinogenic heavy metals (e.g., *ED*_*tot*_ x 365 days/year).

(5) $$\begin{array}{l} TCR=\frac{\left(C_{n}xD_{n}x10^{-3}xCPS_{0}xEF_{r}xED_{tot}\right)}{\left(BW_{a}xAT_{n}\right)} \end{array}$$

where: *CPS*_0_, carcinogenic potential (μg g^−1^ day^−1^).

The effects of HM accumulation in soil, excess uptake by plants, and the risks that HM-contaminated foods can promote to human beings are commonly reported in mining regions (Qing et al., 2015; de Souza et al., 2017; Li et al., 2017). As example, the release and drifting of dust from coal mines in the Qingshui River basin (China) has resulted in pollution of arable soils. Despite the knowledge associated to the deposition of HMs, few studies approach the increase of HM concentration in different edible plant organs cultivated on soils subjected to a long history of animal waste application.

Different environmental agencies have established acceptable levels of HM in food. FAO and EPA-USA established maximum levels for Cu, Zn, Cd, Pb, Cr, and Ni in crop grains of 20, 50, 0.1, 0.2, 1, and 0.04, respectively. However, studies on soils subjected to the addition of urban sludge and animal residues reported increased HM concentration above these limits in grains, fruits, and vegetables (Suarez-Tapia et al., 2017; Zhang et al., 2017). The use of wastewater for irrigation in Iran containing 0.06, 0.010, 0.01, 0.010, and 0.010 mg kg^−1^ of Cu, Zn, Cd, Pb, Cr, and Ni, respectively, caused the accumulation of Cd, Cr, and Pb in wheat and corn grains above the limits established by the EPA. Health risks to adults and especially children by Cu, Cd, and Cr intake in corn and wheat grains were also reported (Asgari and Cornelis, 2015). Animal waste contains HM derived from drugs or feed (Gunkel-Grillon et al., 2015; Couto et al., 2016).

It is worth mentioning that soils with frequent application of organic wastes typically have higher HM concentrations than those described in studies where negative effects of excess HM on edible plant organs and human health risk have been reported, indicating that we might be underestimating the contamination of foods derived from such areas (Table 1). Studies that address the effects of increasing HM concentration in soils subjected to long-term animal waste application and consequent changes of HM concentration in edible plant organs are still scarce. Although organic fertilization recommendations exist both for conventional and organic production systems, the application of organic residues is often carried out indiscriminately in regard to HMs content, increasing their concentration in soils and likely increasing of HM concentration in edible plant organs (Couto et al., 2016; Suarez-Tapia et al., 2017; Zhang et al., 2017). A very important aspect is that in organic production systems, organic residues (including animal manure) are the main—if not the only—source of nutrients for the crops. Considering that organic production systems currently occupy 42 million hectares worldwide (FIBL, 2017), with a global growth rate of 4.5% per year, the risk of HM contamination in the food systems is present, especially in some regions of the world. In Brazil, Japan, and the European Union, the growth rate of the area cultivated under the organic system is 30, 13, and 8% per year, respectively (FIBL, 2017). This emergent risk indicates that the organic residues that will be used as source of nutrients for the crops needs to be assessed in terms of HM concentration.

**Table 1:** Heavy metal contents in soils and vegetables of diverse areas of the world and risks to human health.

**Location**	**Authors**		**As**	**Ba**	**Cd**	**Co**	**Cr**	**Cu**	**Fe**	**Hg**	**Mn**	**Mo**	**Ni**	**Pb**	**Ti**	**V**	**Zn**	**Risks**
Areas with wheat crops	Ran et al., 2016	Total in Soil (HNO_3_, HClO_4_, and HF			1.9			71.1	26.9		435		62.5	16.7			81.9	Cd exhibited high THQ in some areas.
		Total in wheat grain (HNO_3_ and HClO_4_)			1.76			10.3	99.1		106		3.68	0.4			76.4	
Areas grown with vegetables fertigated with wastewater	Singh et al., 2010	Total in Soil (HNO_3_, H2SO_4_, and HClO_4_)			1.92–4.53		17.92–21.18	18.36–25.50					19.93–28.18	14.26–24.10			53.43–64.56	Exhibited high HRI for Cd, Pb, and Ni.
		Total in plant (HNO_3_, H2SO_4_, and HClO_4_)			14 (eggplant)		26 (Bottle gourd)	28 (tomato)					43 (radish)	25 (radish)			85 (Amaranths)	
Mining soil grown with rice and soybean	Silva et al., 2007	Total in soil (HCl and HNO_3_)			20			115	860		536			174			113	High Pb concentrations in soy grains
		Total in grain of Rice(HCl and HNO_3_)			0.4			4.3	15		71			4			59	
		Total in grain of soybean (HCl and HNO_3_)			0.4			11	53		48			8			72	
Fresh wild leafy vegetables in urban and rural areas	Abbasi et al., 2013	Soil (HNO_3_ and HCl)			5		50	30	1100		600			50			60	THQ and HI evidenced adverse/non-carcinogenic health risks. Carcinogenic health risk for Cr and Pb.
		Water cress (HNO_3_ and HClO_4_)			4.2		2.2	4.5	540		25			7.8			48	
		Chicory (HNO_3_ and HClO_4_)			4		11	8	742		23			7			32	
Corn and wheat grown in soil with pig deep-litter	da Rosa Couto et al., 2018	Soil (HNO_3_)	3.7		1.4		11.4	48					4	13			91	Exhibited high HRI and THQ for Cu and Zn.
		Grains of corn(HNO_3_)						3					1				30	
		Grains of wheat (HNO_3_)					1.9	5.7					0.9				95	

In Brazil, the applications of pig slurry, cattle slurry, and pig deep litter for 10 years in sandy soil with low organic matter content under no-till increased Ni, Cu, and Zn concentrations in shoots and grains of corn and wheat (da Rosa Couto et al., 2018). The applications of organic wastes (pig slurry, cattle slurry, and pig deep litter) and mineral fertilizers also increased the values of HRI and THQ for Br and Zn, presenting health risks to adults and especially children who have lower body weight (da Rosa Couto et al., 2018). They also report that Cu concentrations in corn grains of plants grown in soil with application of pig deep litter and cattle slurry were 2.7 and 2.2 mg Cu kg^−1^, respectively. On the other hand, Zn concentrations in corn grains of plants grown in soil with application of pig deep litter, pig slurry, and cattle slurry were 26, 31, and 23 mg Zn kg^−1^, respectively. In the grains of wheat grown in soil with the application of pig deep litter, pig slurry, and cattle slurry, concentrations of Cu were 6.0, 6.0, and 4.5 mg kg^−1^, respectively, and Zn were 96, 95, and 84 mg kg^−1^, respectively. Thus, Cu and Zn concentrations in grains of corn and wheat grown in soil with a long history of application of organic wastes were higher than those found in plants grown in the control soil or even with the application of mineral fertilizer. This justifies the monitoring of concentrations of elements in grains of plants grown in soils with a long history of organic waste application, especially in soils with low capacity for adsorption of elements, such as sandy soils with low organic matter (Brunetto et al., 2014).

Plants have the potential to absorb and accumulate larger amounts of several heavy metals. In studying heavy metal contents in vegetables fertilized with wastewater in India, Singh et al. (2010) found that the concentrations of Cd in plants varied from >2 to 15 mg kg^−1^, while Pb ranged from >1 to 28 mg kg^−1^ and Ni from >1 to 41 mg kg^−1^. The authors verified a risk to consumer health (HRI> 1) through the ingestion of Cd accumulated in radish, cabbage, cauliflower, okra, eggplant wheat and rice; of Pb accumulated in palak, cabbage, cauliflower, Lady's fingers, brinjal, wheat, and rice; and Ni accumulated in palak, cauliflower, wheat, and rice. However, it is important to observe the proportion of vegetables and cereals in the diet, which may change according to the culture of each place and country, causing higher or lower risk.

## Final considerations

The use of waste as a source of nutrients in plant production systems, traditional, and organic, is common worldwide, and important strategy for nutrient cycling. However, long-term application of such waste causes the increase of HM concentrations in soils, increasing HM uptake by plants and assimilation in edible organs such as grains, as indicated by the data presented in Table 1. As different plant organs can be used in the preparation of numerous products for human consumption, it is necessary to monitor the concentrations of HM in edible plant organs of different species and cultivars fertilized with organic waste. This monitoring can be done through indexes such as Igeo, EF, HRI, THQ, TCR, allowing us to estimate the possible dangers of HMs to the health of children, young adults, and adults who eat food derived from plants grown in soils with a history of animal waste application. Thus, we recommend careful consideration of practices that indiscriminately use animal waste in plant production to avoid HM accumulation and health hazards to consumers. Moreover, strongly indicate that evaluation of metal contamination in foods derived from plants cultivated using animal waste should be commonplace, and further studies of how widespread that is should be conducted by the scientific community.

## Author contributions

RdRC: wrote the first draft of the manuscript. MS: organized the database. JC, LG, CC: senior researchers in the field of soil science. They made specific contributions of the area. FR: senior researchers in the field of plant physiology. They made specific contributions of the area. ML: senior researchers in the field of agroecology. They made specific contributions of the area. All authors contributed to the revision of the manuscript, read and approved the version sent.

### Conflict of interest statement

The authors declare that the research was conducted in the absence of any commercial or financial relationships that could be construed as a potential conflict of interest.
